# STX4 as a potential biomarker for predicting prognosis and guiding clinical treatment decisions in clear cell renal cell carcinoma

**DOI:** 10.1016/j.heliyon.2023.e23918

**Published:** 2023-12-21

**Authors:** Kai Zeng, Qinyu Li, Xi Wang, Chaofan Liu, Bingliang Chen, Guoda Song, Beining Li, Bo Liu, Xintao Gao, Linli Zhang, Jianping Miao

**Affiliations:** a Department of Urology, Tongji Hospital, Tongji Medical College, Huazhong University of Science and Technology, Wuhan 430030, Hubei, China; b Department of Urology, the First Affiliated Hospital of Shihezi University, Shihezi 832008, Xinjiang, China; c Department of Oncology, Tongji Hospital, Tongji Medical College, Huazhong University of Science and Technology, Wuhan 430030, Hubei, China; d Department of Urology, Sir RunRun Shaw Hospital, College of Medicine, Zhejiang University, Hangzhou, China; e Department of Geriatrics, Tongji Hospital, Tongji Medical College, Huazhong University of Science and Technology, Wuhan 430030, Hubei, China

**Keywords:** STX4, ccRCC, Drug sensitivity, Immunotherapy, Targeted therapy

## Abstract

Clear cell renal cell carcinoma (ccRCC) represents a frequent subtype of kidney cancer, with the prognosis remaining poor for individuals with metastatic disease. Given its resistance to both radiation and chemotherapy, targeted therapies and immunotherapies have emerged as critical for effective ccRCC treatment. Within this context, the SNARE protein STX4, which is associated with malignant cancer cell migration, provides a promising focus. The underlying mechanism, however, requires further illumination. Furthermore, the influence of STX4 on the ccRCC tumor microenvironment remains to be determined. In our research, we utilized multiple databases and immunohistochemical staining to confirm differential STX4 expression and its prognostic implications. We evaluated the potential tumor-promoting function of STX4 in ccRCC cell lines through molecular studies. Additionally, we conducted functional enrichment analysis to delve deeper into the underlying mechanisms and performed immune infiltration and drug sensitivity analyses to assess the potential of STX4 as a prognostic biomarker and therapeutic target. Our study reveals that STX4 contributes to cancer progression by enhancing AKT expression and stimulating the activation of VEGF signaling pathways. Additionally, STX4 further fosters CD8^+^ T-cell infiltration and diminishes the percentage of CAFs and M2-TAMs. Our findings suggest that patients presenting higher STX4 levels may exhibit enhanced responsiveness to immunotherapy and higher sensitivity to the medications axitinib and everolimus. Finally, we propose STX4 expression assessment as a novel approach to predict patient response to respective immunotherapies and targeted treatments, hence potentially improving patient outcomes.

## 1 Introduction

In recent decades, renal cell carcinoma (RCC) has emerged as a global health concern due to its increasing incidence and resulting status as the primary cause of death among urological malignancies [1]. Clear cell renal cell carcinoma (ccRCC), representing the most common RCC subtype, is particularly challenging to manage [2]. Surgical intervention remains the most efficacious treatment for ccRCC, yet postsurgical relapse and metastatic progression are common and considerably augment the likelihood of cancer mortality [3,4]. Amid continuing advancements in cancer diagnosis and therapy, the long-term survival rate for metastatic ccRCC patients rests at approximately 20 %, highlighting the critical need for more effective treatments [5]. Given the resistance of ccRCC to conventional radiation and chemotherapy, targeted therapeutics and immunotherapeutic interventions have progressively taken center stage in ccRCC patient management strategies [6]. In particular, immune checkpoint blockade (ICB) therapies have reshaped the landscape of cancer treatment [7]. Checkpoint blocking in conjunction with other anticancer medications is now the first-line treatment for advanced ccRCC. Immune suppression is abolished by ICB in a subset of patients with ccRCC, resulting in astounding clinical improvements [8]. However, it remains that a significant number of patients do not qualify for ICB treatment or do not show the desired response, underscoring the persistent need for reliable biomarkers to guide treatment selection.

Cancer metastasis embodies a complex process involving the dispersal of cancer cells from the initial tumor site to distant organs. This process hinges on the intricate, bidirectional interactions between cancer cells and their environment [9]. During metastasis, cancer cells are known to infiltrate the extracellular matrix (ECM) using the mechanism facilitated by invadopodia. This process is considerably influenced by Soluble N-ethylmaleimide-sensitive factor attachment protein receptors (SNAREs) which are essentially involved in intracellular vesicle trafficking [10]. There is substantial evidence from previous research indicating that specific SNAREs, especially those participating in the transport of invadopodium-associated proteins, have key roles in enabling the invasive and migratory characteristics observed in malignant cancer cells [[11], [12], [13]]. Syntaxin 4 (STX4), one member of the SNARE protein family, is implicated in shepherding MT1-MMP to the plasma membrane [14]. Recent studies have indicated that STX4 increases breast tumor and ovarian cancer invasion by mediating invadopodium development [[15], [16], [17]]. The prognostic utility of STX4 in ccRCC has likewise been alluded to in previous studies [18]. Despite these advances, a significant knowledge gap exists regarding the direct impact of STX4 on ccRCC cell proliferation and invasion and its potential influence on the tumor microenvironment.

In the current study, we explored the expression pattern and clinical significance of STX4 in depth. A series of molecular experiments were conducted to evaluate the influence of STX4 on the biological features of ccRCC cells. To identify differences and potential mechanisms between groups with high and low STX4 expression, functional analysis was performed. Furthermore, the correlation between the degree of immune cell infiltration and STX4 expression was assessed through the use of multiple algorithms. To evaluate its potential as a clinical tool to guide treatment selection, we investigated the relationships between patient responses to immunotherapy and targeted therapies and STX4 expression. This comprehensive examination could offer valuable insights into the functions of STX4 and its therapeutic implications in ccRCC management.

## 2 Materials and methods

### 2.1 Data collection and prognosis analysis

We sourced expression profiles and clinical data of KIRC, KIRP, and KICH from The Cancer Genome Atlas (TCGA) database. Additionally, we obtained expression data of an immunotherapy cohort undergoing anti-PD-1 therapy (nivolumab) in RCC from the Gene Expression Omnibus (GEO) database (GSE67501) [19]. We also used the NIHMS1611472 dataset from the supplemental material of a previous study, which included 1006 ccRCC patients treated with either nivolumab or everolimus [20]. To ascertain the prognostic value of STX4, we conducted a Cox regression analysis to evaluate the relationship between STX4 expression and various survival outcomes, specifically overall survival (OS), progression-free survival (PFS), and disease-specific survival (DSS), in ccRCC patients. Furthermore, we examined the correlation between STX4 expression and various clinicopathological characteristics within the TCGA-KIRC cohort.

### 2.2 Functional enrichment and immune cell inﬁltration analyses

Utilizing LinkedOmics, we conducted co-expression analysis for the TCGA-KIRC cohort [21]. The results are depicted in a heatmap that reveals genes exhibiting high positive or high negative correlation with STX4. To further elucidate the functional significance of these co-expressed genes, we applied the "LinkInterpreter" module within LinkedOmics. This tool facilitated Gene Ontology (GO) and Kyoto Encyclopedia of Genes and Genomes (KEGG) enrichment analyses, which shed light on the biological processes, cellular components, molecular functions, and signaling pathways in which these co-expressed genes are implicated.

In this study, patients were divided into two groups, STX4^high^ and STX4^low^, using the median value of STX4 expression as the cutoff. Infiltration estimate data computed by CIBERSORT, TIMER, xCell, quanTIseq, MCP-counter, and EPIC algorithms were downloaded from TIMER 2.0 [22]. The differing levels of immune cell infiltration in both the STX4^high^ and STX4^low^ groups were visualized using a heatmap. Applying the ssGSEA method, we compared the infiltration levels of 28 immune cells between the STX4^high^ and STX4^low^ groups [23]. The immune score and stromal score of each patient were calculated by the “ESTIMATE” package [24]. A comparison of the human leukocyte antigen (HLA) gene family was made between the STX4^high^ and STX4^low^ groups. To guide treatment decisions in clinical practice, we focused our analysis on several immune cells that have been identified to have significant associations with immunotherapy efficacy. We obtained the expression data of three cell types (CAFs, MDSCs, and M2-TAMs), which are believed to inhibit T-cell infiltration in tumors, of TCGA patients from the TIDE database [25]. The comparison of these cells was performed between the STX4^high^ and STX4^low^ groups.

### 2.3 Immunotherapy and targeted therapy response prediction

To predict potential immunotherapy responses among patients with varying STX4 expression levels, we initially assessed the differential expression of immune checkpoint genes between the STX4^high^ and STX4^low^ groups. Additionally, we retrieved the Immunophenoscore (IPS) for ccRCC patients from The Cancer Immunome Database (TCIA). The IPS for each patient was objectively determined by considering four categories of genes that determine immunogenicity: effector cells, immunosuppressor cells, MHC molecules, and immune modulators [26]. Higher IPS scores typically denote elevated immunogenicity. Chemokines and chemokine receptors play vital roles in the orchestration of host immune responses to cancer [27]. Given the integral roles that chemokines and chemokine receptors play in coordinating host immune responses to cancer, we also evaluated the correlation between STX4 expression and the expression of CXCL9 and CXCL10, which have been previously reported to favor the emergence of a "hot" tumor microenvironment [28]. The tumor mutation burden (TMB), which has been identified as a predictive biomarker for determining patients' likelihood of positive response to ICB, was also explored across patients with varying STX4 levels [29]. Finally, we utilized two immunotherapy cohorts (GSE67501 and NIHMS1611472) to validate the identified correlation between STX4 expression and response to immunotherapy.

We also compared the susceptibility to sorafenib, sunitinib, pazopanib, and axitinib across the STX4^high^ and STX4^low^ groups. The half maximal inhibitory concentrations (IC50) of these drugs were compared by applying the “pRRophetic” package [30]. Moreover, RNA-seq and DTP NCI-60 data from the CellMiner database were downloaded, and drugs with FDA approval or those undergoing clinical trials were selected for further analysis. The correlation between STX4 expression and drug sensitivity was estimated.

### 2.4 Renal cell carcinoma cell lines and tissue specimens

Two ccRCC cell lines (786O and OSRC-2) were procured from the CAS Cell Bank (Shanghai, China). These cells were cultured in RPMI 1640 medium, supplemented with 10 % FBS (Boster Biological Technology, Wuhan, China). Standard cell culture conditions were maintained at a temperature of 37 °C in an incubator with a CO2 concentration of 5 %. Tissue samples, both tumorous and matched normal, were obtained from patients with ccRCC who had undergone either partial or radical nephrectomy procedures at Tongji Hospital. The Institutional Research Ethics Committee of Tongji Hospital provided the requisite ethical approval for this study (TJ-IRB-20230331).

### 2.5 SiRNA transfection

siRNA and corresponding negative control were synthesized by RiboBio Company (Guangzhou, China). For the process of transfection, Lipofectamine 3000 (Thermo Fisher Scientific) was employed, wherein OSRC-2 and 786-O cells were transfected with STX4 or control siRNA at a concentration of 80 nmol/L. Post transfection, we determined the efficacy of the knockdown by employing qRT-PCR and Western Blot analysis. These analytical methods were executed as described in our previous studies [31].

### 2.6 CCK-8 and EdU assays

Cells were plated at a density of 2000 cells/well in 96-well plates, and cell viability and cytotoxicity were measured according to the protocol (Yeasen Biotechnology, Shanghai, China). When performing the drug sensitivity analysis, the cells were incubated with 100 μL of fresh medium containing vehicle (DMSO) or drugs for 48 h and were assayed for cell viability measurement. The following drugs were used at the indicated concentrations: axitinib (1 μM, Selleck, S1005), sunitinib (5 μM, Selleck, S7781), pazopanib (5 μM, Selleck, S3012) and everolimus (25 nM, Selleck, S1120). For the EdU assays, post transfection for 48 h, cells were seeded in 12-well plates at a density of 8 × 10^4^ cells per well. The cells were then incubated with a medium containing 50 μM EdU for a period of 2 h. Subsequently, the cells were fixed with 4 % paraformaldehyde at room temperature for a duration of 20 min. This was followed by staining the cells with Hoechst reagent and Apollo staining solution. The percentage of EdU-positive cells was calculated under a microscope in five randomly selected fields.

### 2.7 Wound-healing and transwell assays

Transfected 786O or OSRC-2 cells were seeded into 6-well plates at a cellular density of 2 × 10^5^ cells per well. Upon reaching 90 % confluence of the cell monolayers, a scratch was made parallely using a 1 ml pipette tip to simulate a wound. The healing rate of this wound was subsequently assessed after the 24 h using ImageJ software. For the transwell assay, 3 × 10^4^ cells were cultured in serum-free medium in the upper chamber, while 500 μl of 10 % FBS-containing medium was added to the bottom chamber. After 24 h, the pierced cells were fixed in 4 % paraformaldehyde and stained with 0.1 % crystal violet. Under a microscope, cells were counted in five randomly selected fields.

### 2.8 Flow cytometry analysis of the cell cycle and apoptosis

To ascertain the rate of apoptosis, harvested cells were twice rinsed with PBS. These were then subjected to co-staining with PI and Annexin V- FITC (Yeasen Biotechnology, China), followed by an incubation period of 10–15 min before flow cytometry detection. To study the effect of everolimus between the STX4^high^ and STX4^low^ groups, transfected 786O and OSRC2 cells were incubated with vehicle (DMSO) or everolimus (25 nM, Selleck, S1120) for 48 h, and the proportion of apoptotic cells was detected. For the analysis of cell cycle distribution, collected cells were treated with RNase and PI (Yeasen Biotechnology, China) for 30 min at 4 °C after overnight fixation in 70 % ethanol. A CytoFlex cytometer was used to obtain flow cytometry data, which were then analyzed using FlowJo V10 software.

### 2.9 Immunohistochemical (IHC) staining

Immunohistochemical staining was carried out on paraffin-embedded sections derived from clinical specimens of our hospital. We utilized rabbit anti-STX4 primary antibody (A5996; ABclonal) and goat anti-rabbit secondary antibodies (GB23303; Servicebio) for this purpose. A DAB kit was employed to visualize the antibody interactions subsequent to incubation with the secondary antibodies.

### 2.10 Pan-cancer analysis of STX4

To elucidate the expression differences across 33 cancer types, we utilized the TIMER database. The RNA-seq data for these cancer types was downloaded from the TCGA database, which allowed us to evaluate the correlation between STX4 expression and immune checkpoint genes. Additionally, univariate Cox regression analysis was performed to explore the relationship between STX4 expression and OS and PFS in these 33 cancer types. Finally, we conducted correlation analysis between TMB, MSI, and STX4 expression across 33 cancer types.

### 2.11 Statistical analysis

Data analyses were performed using R (version 4.0.4) and GraphPad Prism (version 7). The Spearman correlation method was employed to compute correlations. Two groups were compared using Student's t-test or Wilcoxon test. For comparison across multiple groups, we utilized the Kruskal-Wallis test and One-Way Analysis of Variance (ANOVA). A P-value less than 0.05 was considered indicative of statistical significance.

## 3 Results

### 3.1 STX4 is positively correlated with poor prognosis in ccRCC

**Fig. 1:**
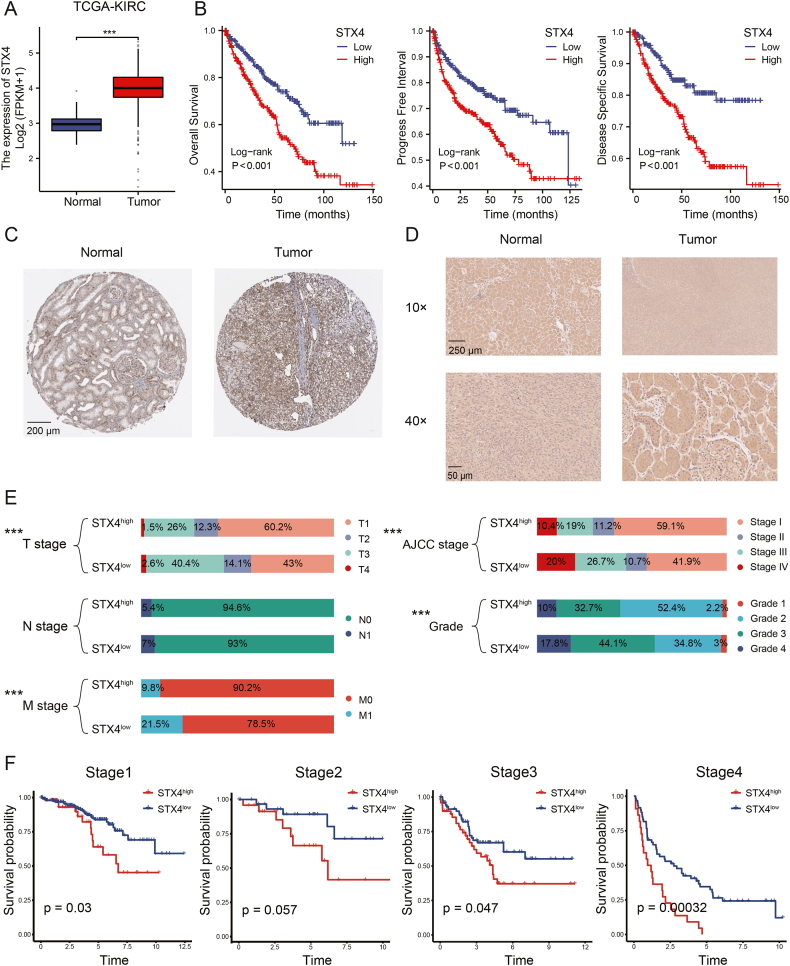
STX4 expression is enhanced in ccRCC and associated with worse prognosis. (A) Expression of STX4 between tumor and paired normal tissues in TCGA-KIRC cohort. (B) Differences in OS, PFS, and DSS between STX4^high^ and STX4^low^ patients in the TCGA-KIRC cohort. (C–D) Immunohistochemical staining for STX4 expression between tumor and normal tissues in the HPA database (C) and specimens collected in Tongji Hospital (D). (E) Differences in clinical parameters between STX4^high^ and STX4^low^ patients. (F) Survival difference between STX4^high^ and STX4^low^ ccRCC patients in different pathologic stages.

From the TCGA database, we observed a significant increase in STX4 expression in ccRCC (Fig. 1A). Patients demonstrating high STX4 expression had notably reduced OS, PFS, and DSS compared to those with low STX4 expression (Fig. 1B). We further investigated the role of STX4 in other subtypes of RCC. Interestingly, we found that STX4 expression was notably upregulated in KIRP but downregulated in KIRP (Figs. S1A and B). Deviating from its impact in KIRC, STX4 did not present a significant association with the prognosis of KIRP and KICH (Figs. S1C and D). Additionally, we reinforced the upregulation of STX4 in ccRCC by cross-validating our findings with the HPA database and IHC staining, which yielded concordant results (Fig. 1C and D). As anticipated, patients within the STX4^high^ subgroup exhibited a higher proclivity toward clinical advancement (Fig. 1E). Intriguingly, with progressing pathological stages, the role of STX4 appeared to escalate in the survival trajectory of patients (Fig. 1F). Cumulatively, these findings suggest that STX4 may function as a tumor enhancer in ccRCC.

### 3.2 Functional enrichment analyses of STX4

**Fig. 2:**
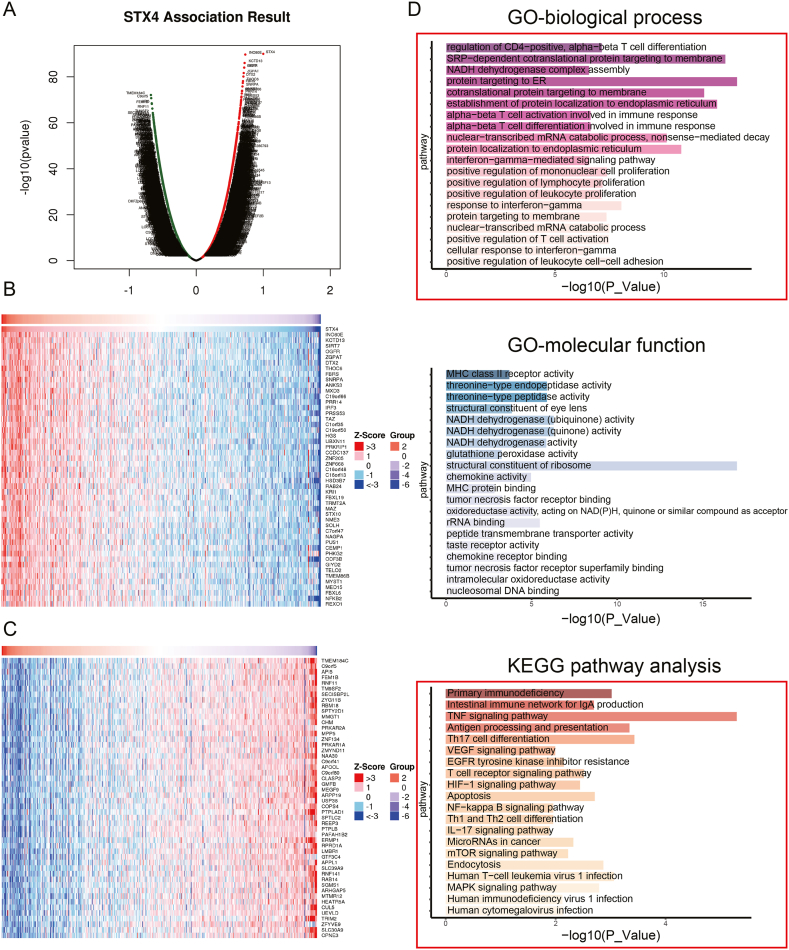
The co-expression networks and functional enrichment analysis of STX4 in ccRCC. (A) Volcano plot of the genes significantly correlated with STX4. (B–C) Heatmap of the top 50 genes positively (B) and negatively (C) related to STX4. (D) Significantly enriched GO terms and KEGG pathways associated with STX4.

To gain a deeper understanding of the biological functions of STX4, we conducted a co-expression analysis in the TCGA-KIRC cohort using LinkedOmics. A volcano plot depicted genes that are correlated with STX4 (Fig. 2A), and the top 50 positively and negatively correlated genes were visualized by a heatmap (Fig. 2B). Functional enrichment analysis revealed a strong correlation between STX4 and immune activation, immune cell proliferation, and the overall immune response (Fig. 2C). Interestingly, we also found that STX4 is associated with several pathways that play significant roles in ccRCC progression, including the VEGF signaling pathway, mTOR signaling pathway, NF−κB signaling pathway, MAPK signaling pathway, and HIF−1 signaling pathway (Fig. 2C).

### 3.3 Knockdown of STX4 suppresses ccRCC proliferation, invasion, and metastasis

**Fig. 3:**
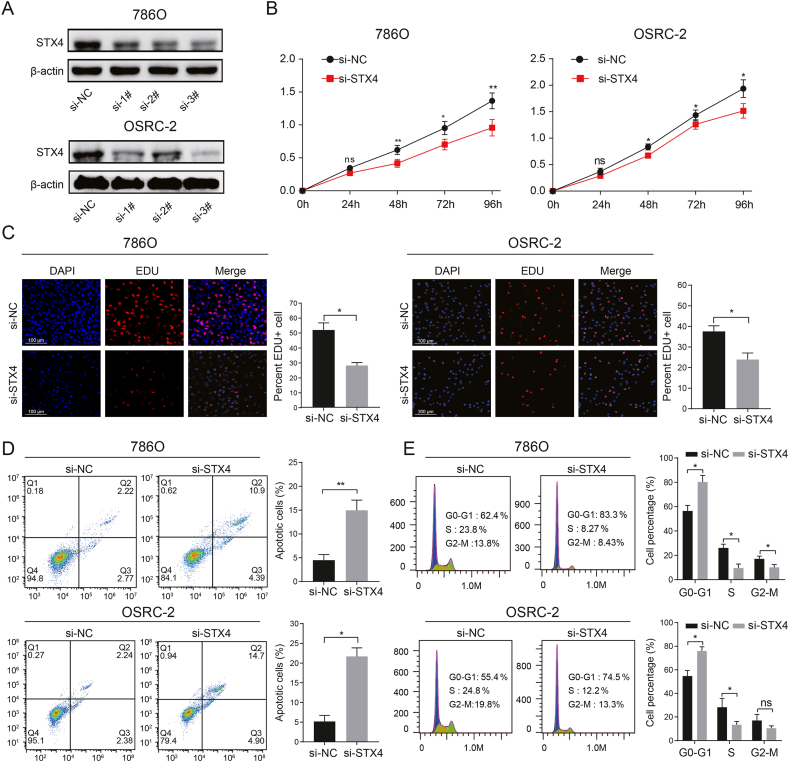
Downregulation of STX4 represses proliferation and increases apoptosis in ccRCC. (A) The knockdown of STX4 detected by Western blot. (B–C) Cell proliferation between the si-NC and si-STX4 groups determined by CCK-8 and EdU assays in ccRCC cells. (D–E) Apoptosis rate and cell cycle estimated by flow cytometry in OSRC-2 and 786-O cells. (*p < 0.05, **p < 0.01).

**Fig. 4:**
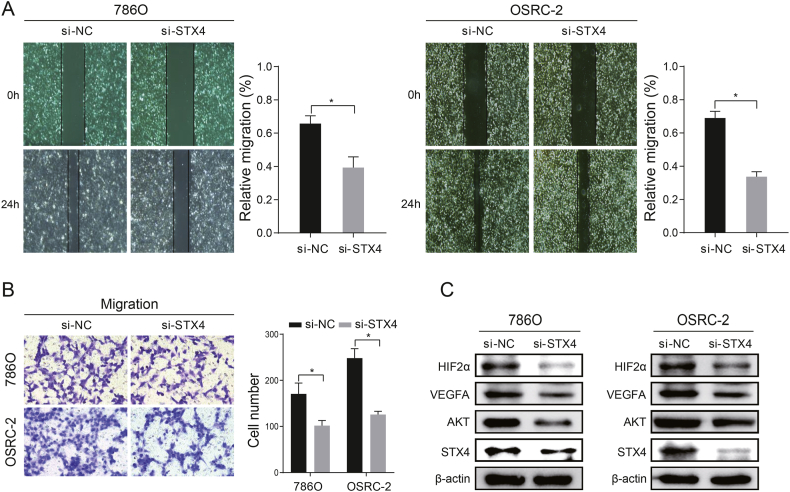
**STX4 regulated the migration ability of ccRCC cells** (A–B) The migration ability of ccRCC cells was determined by wound healing and transwell assays in the si-NC and si-STX4 groups. (C) Detection of AKT, HIF2α, and VEGFA expression in 786-O and OSRC-2 cells after STX4 knockdown.

We further explored the potential pro-oncogenic effect of STX4 in 786O and OSRC-2 cells. After transfecting cells with siRNA to downregulate STX4, we confirmed the decrease in STX4 levels through qRT-PCR (Fig. S2) and Western blotting (Fig. 3A). Cell proliferation was evidently suppressed in cells with STX4 knockdown, as indicated by the CCK-8 and EdU assays (Fig. 3B and C). Simultaneously, cell apoptosis was increased due to STX4 knockdown (Fig. 3D). Additionally, the reduction in STX4 levels also notably triggered cell cycle arrest at the G0/G1 phase (Fig. 3E). Furthermore, the wound healing and Transwell assays suggested a sharp decrease in cell migration ability with STX4 downregulation (Fig. 4A and B). Interestingly, upon conducting Western blot analysis, we observed a decrease in the expression of AKT, HIF2α, and VEGFA following STX4 knockdown. This alteration might provide an explanation for the suppression of ccRCC proliferation and metastasis (Fig. 4C, S3).

### 3.4 STX4 promotes the infiltration of CD8^+^ T cells and reduces the proportions of CAFs and M2-TAMs

**Fig. 5:**
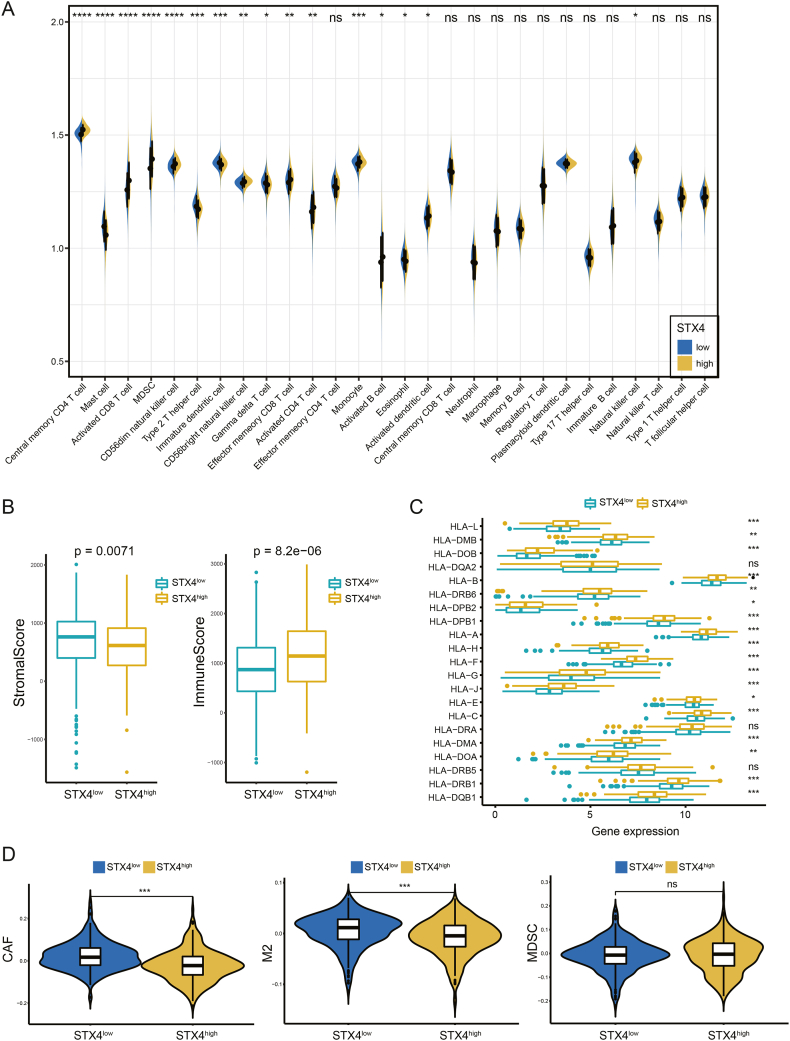
**Association between the characteristics of the immune microenvironment and STX4**. (A) The immune cell infiltration level between the STX4^high^ and STX4^low^ groups. (B) Comparison of the immune score and stromal score between the STX4^high^ and STX4^low^ groups. (C) Comparison of HLA gene family expression between the STX4^high^ and STX4^low^ groups. (D) The infiltration levels of CAFs, M2-TAMs, and MDSCs in the STX4^high^ and STX4^low^ groups. (*p < 0.05, **p < 0.01, ***p < 0.001).

To characterize the immune status, we carried out a comparison of immune cell infiltration levels between the STX4^high^ and STX4^low^ groups within the TCGA-PRAD cohort. Most algorithms revealed that patients with high STX4 expression had increased immune cell inﬁltration levels, especially for T cells (Fig. 5A, S4). Notably, the infiltration levels of CD8^+^ T cells, CD4^+^ T cells, neutrophils, and dendritic cells were associated with altered STX4 gene copy numbers (Fig. S5). We calculated the ImmuneScore and StromalScore, and those in the STX4^high^ group reflected lower stromal activity and increased immune activity (Fig. 5B). Furthermore, we noticed that the expression levels of most HLA-related genes, which play a pivotal role in antigen presentation during immune recognition processes, were also elevated in the STX4^high^ groups (Fig. 5C).

**Fig. 6:**
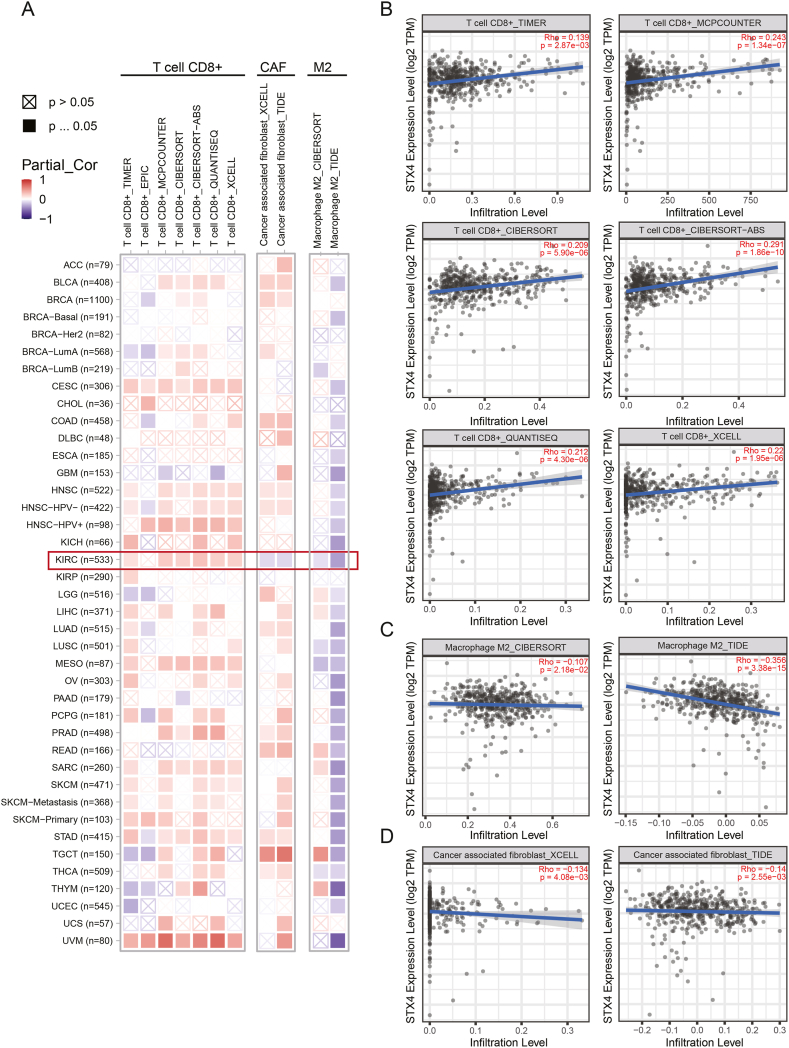
Correlation between STX4 expression and immune infiltration levels of CD8^+^ T cells, CAFs, and M2-TAMs. (A) Multiple algorithms were used to assess the relationship between STX4 expression and immune infiltration levels of CD8^+^ T cells, M2-TAMs, and CAFs. (B–D) Corresponding correlation analysis.

To help accurately predict the effectiveness of immunotherapy, we compared the infiltration levels of CAFs, MDSCs, and M2-TAMs. Patients with high expression of STX4 had considerably fewer CAFs and M2-TAMs (Fig. 5D). We further validated the association between STX4 expression and CD8^+^ T cells, CAFs, and M2-TAMs through TIMER 2.0. STX4 expression was positively associated with CD8^+^ T-cell infiltration and negatively associated with M2-TAM infiltration in a majority of cancers (Fig. 6A). The respective correlation coefficients are shown in Fig. 6B and C. Notably, while STX4 expression exhibited a positive correlation with CAFs in other types of tumors, it displayed a negative correlation with CAF infiltration in ccRCC (Fig. 6D).

### 3.5 STX4 as a potential biomarker to predict therapeutic benefits

**Fig. 7:**
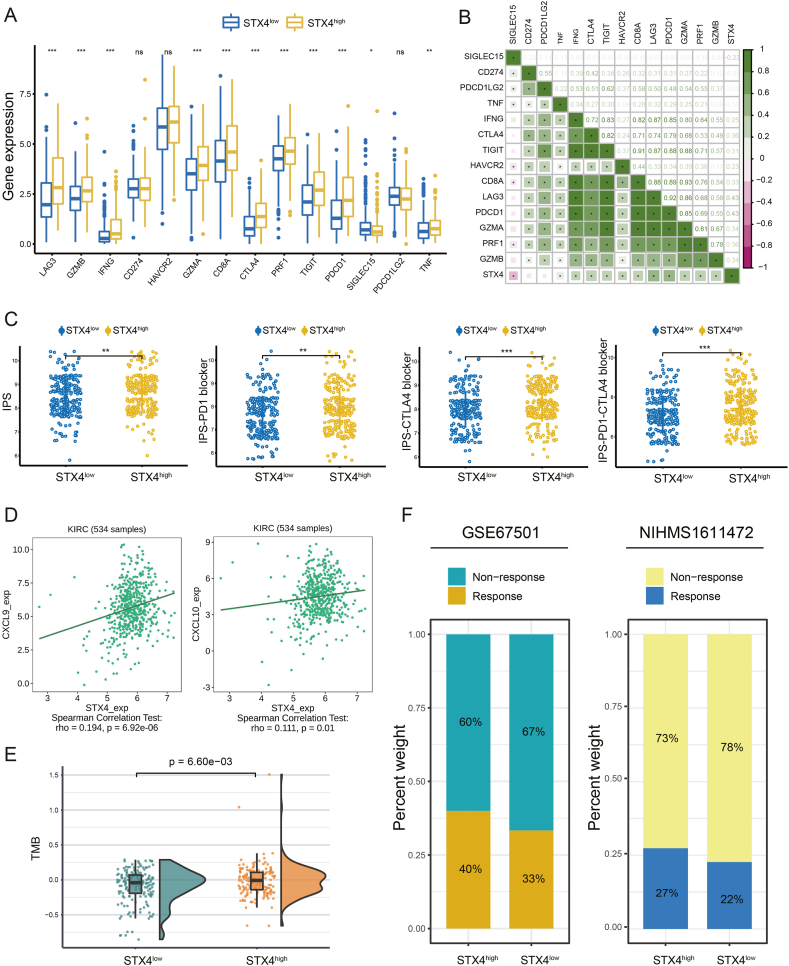
STX4 contributes to predicting immunotherapy efficiency. (A) Comparison of the expression of immune checkpoint genes between the STX4^high^ and STX4^low^ groups. (B) The correlation between STX4 expression and immune checkpoint gene expression. (C) The IPS score between the STX4^high^ and STX4^low^ groups. (D) The correlation between STX4 expression and CXCL9 and CXCL10 expression in the TISIDB database. (E) The difference in TMB between the STX4^high^ and STX4^low^ groups. (F) The proportion of response rate in the two immunotherapy cohorts receiving anti-PD-1 treatment between the STX4^high^ and STX4^low^ groups. (*p < 0.05, **p < 0.01, ***p < 0.001).

To assess the relationship between immunotherapy response and STX4 expression, we evaluated the correlation between STX4 and immune checkpoint gene expression. Crucially, the expression of most immune checkpoint genes, including CTLA4 and PD-1, was found to be higher in the STX4^high^ group than in the STX4^low^ group (Fig. 7A). The correlation analysis suggested a positive association between STX4 expression and immune checkpoint gene expression (Fig. 7B). Using IPS analysis to assess the immunogenicity of the two prognostic groups, we found that patients with high STX4 expression scored higher on IPS, IPS-CTLA4, IPS-PD1, and IPS-PD1-CTLA4, indicating a potentially increased response to immunotherapy for these patients (Fig. 7C). In addition, analysis using the TISIDB database to determine correlations with various chemokines (Fig. S6) showed that STX4 also had a positive correlation with CXCL9 and CXCL10 (Fig. 7D), which contribute to a "hot" tumor microenvironment and can increase immunotherapy effectiveness. TMB is emerging as a promising biomarker for predicting patients' ICB responses [32]. We discovered that patients in the STX4^high^ group had a higher TMB than those with low expression of STX4 (Fig. 7E) and that STX4 expression was positively related to TMB (Fig. S7). In summary, all our results indicate that patients expressing high levels of STX4 may benefit more from immunotherapy. These findings were further confirmed when evaluated in two independent cohorts undergoing immunotherapy (GSE67501 and NIHMS1611472), where a higher response rate to immunotherapy was observed in STX4^high^ patients (Fig. 7F).

**Fig. 8:**
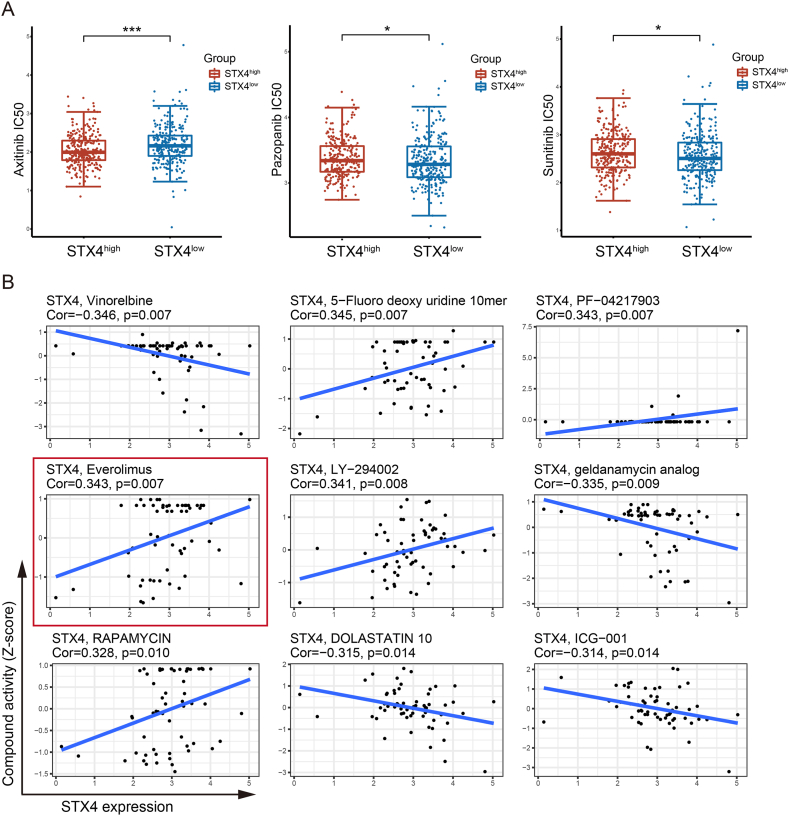
STX4 predicts the therapeutic benefits of targeted therapies. (A) The estimated IC50 values of axitinib, pazopanib, and sunitinib between the STX4^high^ and STX4^low^ groups. (B) Correlation of compound activity (Z score) and STX4 expression. (*p < 0.05, **p < 0.01, ***p < 0.001).

**Fig. 9:**
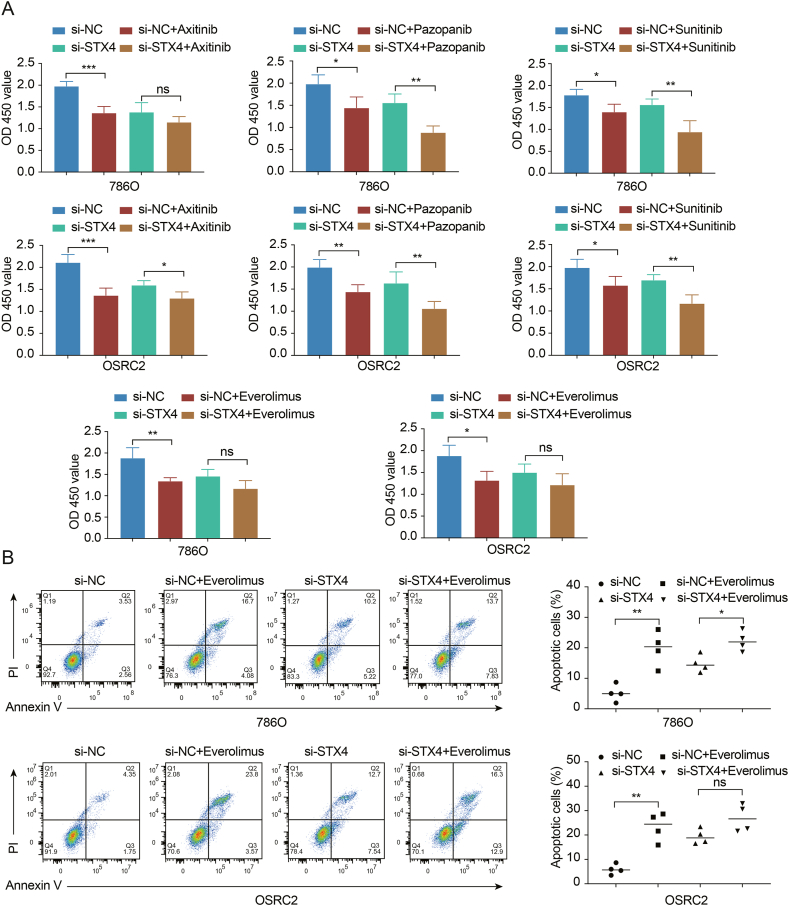
Drug sensitivity analysis (A) CCK-8 assays were used to measure axitinib (1 μM), pazopanib (5 μM), sunitinib (5 μM), and everolimus (25 nM) sensitivity of 786O or OSRC2 cells after knockdown of STX4 for 48 h. (B) The percentage of apoptotic cells with or without knockdown of STX4 in the absence or presence of everolimus (25 nM) was analyzed by flow cytometry assays.

Given that tyrosine kinase inhibitors remain the first-line therapeutic choice for advanced ccRCC, we compared the sensitivity of sorafenib, sunitinib, pazopanib, and axitinib across the STX4^high^ and STX4^low^ groups. The patients in the STX4^high^ group were more likely to respond to axitinib, while the patients with low expression of STX4 responded better to sunitinib and pazopanib (Fig. 8A). For sorafenib, the difference between the two groups was not statistically significant (Fig. S8). By analyzing the CellMiner database, we identified 37 drugs with sensitivities significantly related to STX4 expression (Table S1). Interestingly, everolimus, an mTOR pathway inhibitor approved for the treatment of refractory RCC [33,34], showed a positive correlation with STX4 expression (Fig. 8B). Patients expressing high STX4 levels also had higher sensitivity to everolimus. To further validate these findings, we compared cell viability between cell groups treated with si-STX4 and si-NC following treatment with these drugs. Surprisingly, consistent with the results of the bioinformatics analysis, both the therapeutic effects of axitinib and everolimus were diminished following STX4 knockdown (Fig. 9A). Furthermore, everolimus-induced apoptotic cells were significantly less prevalent in the si-STX4 group than in the si-NC group (Fig. 9B). Nevertheless, it seemed that there was no close relationship between STX4 expression and the drug sensitivity of sunitinib and pazopanib in ccRCC (Fig. 9A).

### 3.6 Pan-cancer analysis

**Fig. 10:**
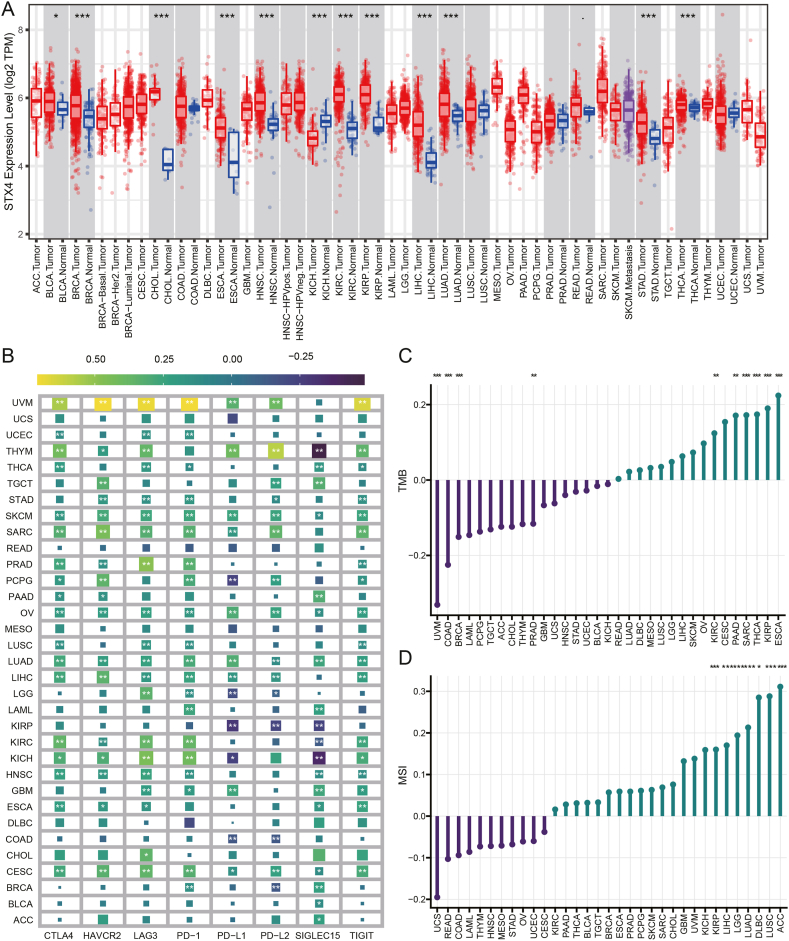
A pan-cancer analysis of STX4. (A) The expression of STX4 in various cancers analyzed by the TIMER database. (B–D) Correlation between immune checkpoint genes, TMB, and MSI with STX4 in pan cancers. (*p < 0.05, **p < 0.01, ***p < 0.001).

The TIMER database provided us with a visualization of the differential expression of STX4 in a range of cancers. Differential expression of STX4 was observed in BLCA, BRCA, CHOL, ESCA, HNSC, KICH, KIRC, KIRP, LIHC, LUAD, STAD, and THCA (Fig. 10A). Moreover, STX4 was found to be strongly associated with immune checkpoint genes across most tumors (Fig. 10B), and it correlated with TMB and MSI in several cancers (Fig. 10C and D). From survival analysis, we also identified that STX4 expression was linked to OS or PFS in numerous types of tumors (Fig. S9).

## 4 Discussion

Currently, continuous progress in understanding renal cell tumor biology has resulted in the development of molecular therapies aimed at the VEGF and mTOR pathways [35,36]. Concurrently, the emergence of immune checkpoint inhibitors has added another therapeutic option for patients with advanced ccRCC [[37], [38], [39]]. Notably, immunotherapies such as nivolumab have already been incorporated into treatment regimens for metastatic ccRCC [40]. However, a key challenge remains in dealing with the high heterogeneity of tumors, as they vary greatly in their cell biological characteristics and genetic makeup. Consequently, patients suffering from metastatic or advanced RCC often have a poor prognosis, given the lack of effective therapies that can provide long-lasting responses [41,42]. Therefore, it is crucial to focus on identifying and validating reliable biomarkers. These could improve the selection process for first-line treatments and make them more effective by tailoring them more specifically to the individual patient's genetic and biological context.

The process of cancer cell metastasis relies on the delivery of ECM and integrins to the cell surface, driven by various SNARE proteins [43]. These proteins play a critical role in vesicle transport at both the cell surface and within intracellular compartments [43]. STX4 is involved in the formation of cell invadopodium and tumor cell infiltration [16]. A previous study by He et al. suggested the prognostic value of STX4 in kidney renal clear cell carcinoma (KIRC) [18]. Nevertheless, whether STX4 impacts the proliferative and invasive abilities and tumor microenvironment of ccRCC remains to be investigated. In this study, we demonstrated the aberrant upregulation of STX4 expression in ccRCC and discovered its close correlation with the prognosis of ccRCC patients. Interestingly, we observed that STX4 was abnormally overexpressed during tumor progression. Patients in the STX4^high^ group had a higher proportion of advanced clinicopathological characteristics. These findings suggest that STX4 plays a crucial role in the prognosis of patients with advanced and metastatic ccRCC. To decipher the underlying molecular mechanisms, we performed functional enrichment analysis in the STX4^high^ and STX4^low^ groups. This revealed that dysregulation of STX4 was connected with several known cancer-promoting pathways, including VEGF, NF−kappa B, and HIF−1 signaling pathways. Additionally, in 786O and OSRC-2 cells, we conducted molecular experiments to gauge the potential tumor-promoting function of STX4. The results illustrated that STX4 knockdown decreased the proliferation and migratory abilities of ccRCC cell lines and increased apoptosis. Western blot results further suggested that STX4 might impact the levels of AKT, HIF2α, and VEGFA, thereby affecting ccRCC development.

The results of pathway enrichment analysis suggested a link between STX4 and immune responses. This implies potentially significant immunological differences between ccRCC patients with high and low STX4 expression. We observed higher levels of immune cell infiltration, particularly of T cells, in patients showing increased STX4 expression. Several algorithms have confirmed the positive correlation between STX4 expression and infiltration levels of CD8^+^ T cells, which are vital for host defense against tumors [44]. In contrast, we observed a significant reduction in the cell populations of CAFs and M2-TAMs, cells known for their immune-suppressive properties in the tumor microenvironment. Moreover, the expression levels of immune checkpoint genes and the majority of HLA-related genes were considerably elevated in the STX4^high^ group. Higher IPS scores are related to increased immunogenicity [45], and we indeed discovered higher IPS, IPS-CTLA4, IPS-PD1, and IPS-PD1-CTLA4 scores in patients with high STX4 expression. CXCL9 and CXCL10 expression have been reported to contribute to the generation of a "hot" tumor microenvironment [28]. By using the TISIDB database, we identified a positive correlation between STX4 expression and CXCL9 and CXCL10 expression. Recent studies have reported that tumor mutation burden (TMB) correlates with immunotherapy response because it reflects the total neoantigen load [46,47]. Here, we found a strong positive association between STX4 expression and TMB. Patients with high STX4 expression had a higher TMB, indicating a potentially superior response to treatment. These results all suggest that patients with high STX4 expression may benefit from immunotherapy. To validate these findings, we utilized two cohorts of ccRCC patients treated with immunotherapy. Consistent with our previous findings, patients in the STX4^high^ group had a higher immunotherapy response rate.

The European Association of Urology recommends the usage of combination therapies such as nivolumab and ipilimumab or pembrolizumab and axitinib as initial lines of treatment for intermediate and high-risk patients [48]. Another clinical trial reported that the PFS was noticeably extended with the administration of avelumab in combination with axitinib, as compared to sunitinib in patients suffering from advanced RCC [49]. Moreover, targeted therapies for metastatic RCC have been observed to exhibit immunomodulatory effects, including the enhancement of tumor cell antigenicity and incitement of T-cell infiltration [50]. Consequently, the combination of targeted therapies and personalized immunotherapy might be a more suitable therapeutic approach for individuals diagnosed with advanced ccRCC. Thus, we undertook a comparative analysis of the IC50 values of first-line therapeutic agents available in the GDSC database between the groups presenting high and low levels of STX4 expression. Our findings suggest that patients in the STX4^high^ group were more likely to be responsive to axitinib. Everolimus has been approved by the FDA for the treatment of RCC refractory to inhibitors of VEGF receptor signaling [34]. Through analysis results from the CellMiner database, we observed that patients with high expression of STX4 are more sensitive to everolimus, which was preliminarily proven by CCK8 and flow cytometry results. Based on these observations, we posit that STX4 could serve as a biomarker potentially aiding in the selection of treatment regimens in clinical practice. Patients with high STX4 expression appear to show a better response to immunotherapy and increased sensitivity to axitinib and everolimus.

Although we validated the pro-oncogenic effect of STX4 on ccRCC cell lines and identified STX4 as a potential biomarker to aid treatment decisions in ccRCC, several limitations to this study need to be acknowledged. First, animal experiments are vital to further validate the biological functions of STX4 in ccRCC. Additionally, although we validated the correlation between STX4 and immune checkpoint genes, TMB, and MSI by pan-cancer analysis, the specific mechanism still needs further investigation.

## 5 Conclusions

In conclusion, we validated the disparity in the expression of STX4 and its prognostic value in ccRCC. In vitro tests were conducted to examine the cancer-promoting role of STX4 in ccRCC. Additionally, we evaluated the correlations between patient response to immunotherapy, targeted therapies, and STX4 expression. Our results suggest that ccRCC patients with high STX4 expression could potentially benefit from axitinib, everolimus, and immunotherapy. This finding adds to our understanding of STX4 as a promising biomarker for predicting treatment responses and aiding therapeutic decisions in ccRCC.

## Data availability statement

Data will be made available on request, and all the data can be obtained by contacting the corresponding author.

## CRediT authorship contribution statement

**Kai Zeng:** Writing – review & editing, Investigation. **Qinyu Li:** Writing – review & editing, Investigation. **Xi Wang:** Writing – review & editing, Writing – original draft. **Chaofan Liu:** Writing – review & editing, Software. **Bingliang Chen:** Writing – review & editing, Resources. **Guoda Song:** Writing – review & editing, Software. **Beining Li:** Writing – review & editing, Resources. **Bo Liu:** Writing – review & editing, Resources. **Xintao Gao:** Project administration, Conceptualization. **Linli Zhang:** Project administration, Conceptualization. **Jianping Miao:** Writing – review & editing, Project administration, Conceptualization.

## Declaration of competing interest

The authors declare that they have no known competing financial interests or personal relationships that could have appeared to influence the work reported in this paper.
