# Supplementary material for: STX4 as a potential biomarker for predicting prognosis and guiding clinical treatment decisions in clear cell renal cell carcinoma
Source: Heliyon. 2023 Dec 21;10(1):e23918. doi: 10.1016/j.heliyon.2023.e23918 (PMC10788513; doi:10.1016/j.heliyon.2023.e23918)
Supplement: Multimedia component 1 [file mmc1.docx]

| Gene | Drug | cor | pvalue |
| --- | --- | --- | --- |
| STX4 | Vinorelbine | -0.3461273 | 0.006749 |
| STX4 | 5-Fluoro deoxy uridine 10mer | 0.345272407 | 0.006896 |
| STX4 | PF-04217903 | 0.343215502 | 0.00726 |
| STX4 | Everolimus | 0.342696244 | 0.007354 |
| STX4 | LY-294002 | 0.34096319 | 0.007677 |
| STX4 | geldanamycin analog | -0.334535905 | 0.008987 |
| STX4 | RAPAMYCIN | 0.328171951 | 0.010471 |
| STX4 | DOLASTATIN 10 | -0.314596251 | 0.014361 |
| STX4 | ICG-001 | -0.314226651 | 0.014482 |
| STX4 | JNJ-38877605 | 0.312452667 | 0.015077 |
| STX4 | Vinblastine | -0.310254023 | 0.015842 |
| STX4 | Temsirolimus | 0.30470587 | 0.017924 |
| STX4 | Paclitaxel | -0.302867805 | 0.018663 |
| STX4 | Rapamycin | 0.299590524 | 0.020046 |
| STX4 | TAK-901 | -0.296080555 | 0.021622 |
| STX4 | Des-fluoro-TAK-960 | -0.296068798 | 0.021627 |
| STX4 | OSI-027 | 0.29419498 | 0.022511 |
| STX4 | Depsipeptide | -0.292034486 | 0.023568 |
| STX4 | BI-2536 | -0.291373481 | 0.023899 |
| STX4 | Dinaciclib | -0.287837494 | 0.02574 |
| STX4 | Deforolimius | 0.285585188 | 0.026974 |
| STX4 | AMG-458 | 0.282469083 | 0.028764 |
| STX4 | BP-1-102 | -0.281827948 | 0.029144 |
| STX4 | Actinomycin D | -0.2806672 | 0.029843 |
| STX4 | SNS-314 | 0.275622763 | 0.033046 |
| STX4 | Cpd-401 | 0.275445839 | 0.033163 |
| STX4 | PF-04691502 | 0.271681762 | 0.035743 |
| STX4 | EMD-534085 | -0.271545088 | 0.035839 |
| STX4 | Methylprednisolone | 0.261999605 | 0.043153 |
| STX4 | Vincristine | -0.260945934 | 0.044031 |
| STX4 | Mithramycin | -0.257635418 | 0.046884 |
| STX4 | Rabusertib | 0.257587164 | 0.046927 |
| STX4 | Fludarabine | 0.256977842 | 0.047469 |
| STX4 | Amuvatinib | 0.255490623 | 0.048813 |
| STX4 | Volasertib | -0.25508251 | 0.049187 |
| STX4 | VINORELBINE | -0.25444669 | 0.049774 |

Table S1: Drugs significantly associated with the expression of STX4
