# Supplementary material for: STX4 as a potential biomarker for predicting prognosis and guiding clinical treatment decisions in clear cell renal cell carcinoma
Source: Heliyon. 2023 Dec 21;10(1):e23918. doi: 10.1016/j.heliyon.2023.e23918 (PMC10788513; doi:10.1016/j.heliyon.2023.e23918)
Supplement: Multimedia component 2 [file mmc2.pdf]

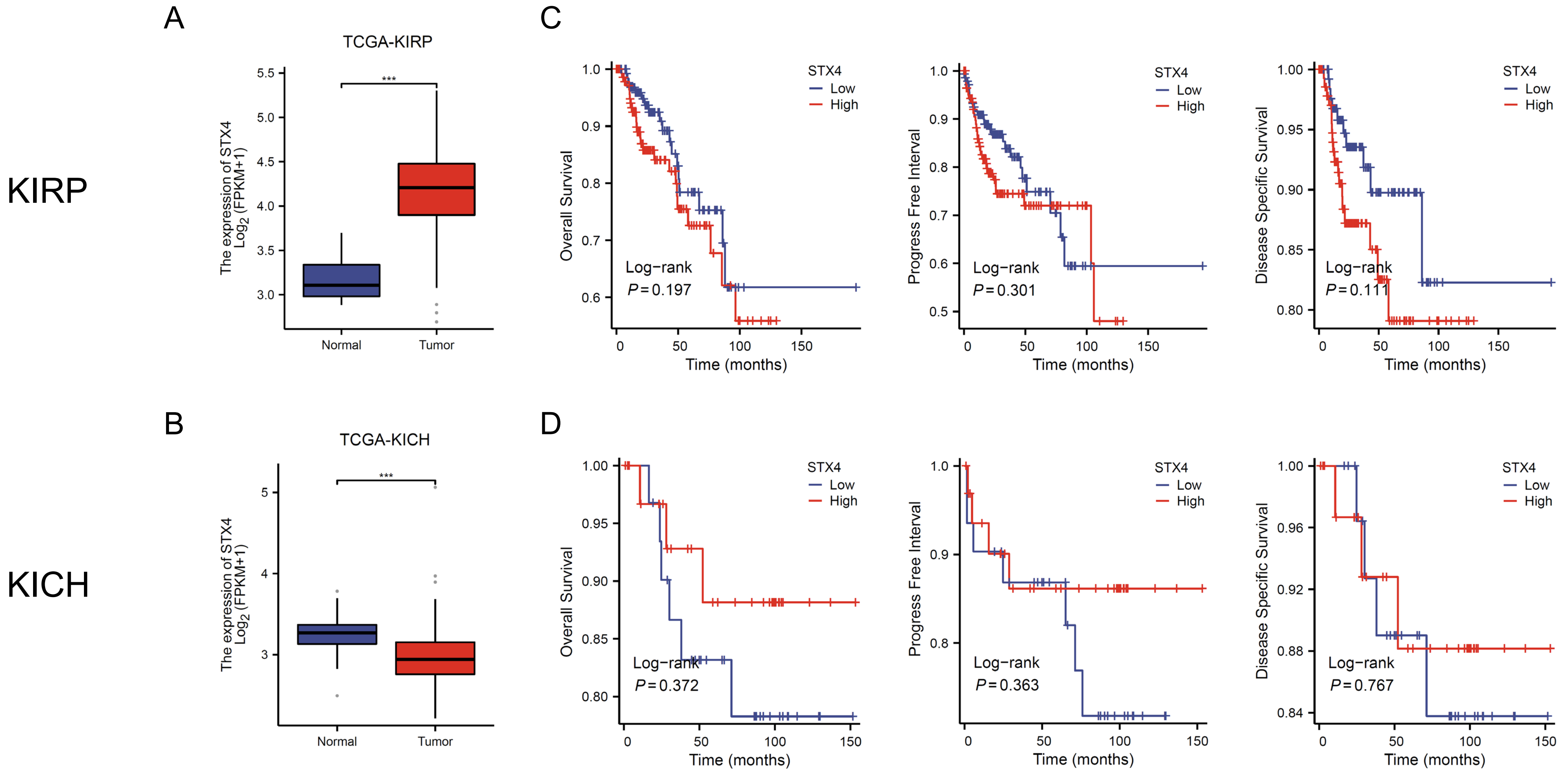

Figure S1: (A-B) Expression of STX4 between tumor and paired normal tissues in TCGA-KIRP (A) and TCGA-KICH (B) cohort. (C-D) Differences in OS, PFS, and DSS between STX4-high and STX4-low patients in TCGA-KIRP (C) and TCGA-KICH (D) cohort.

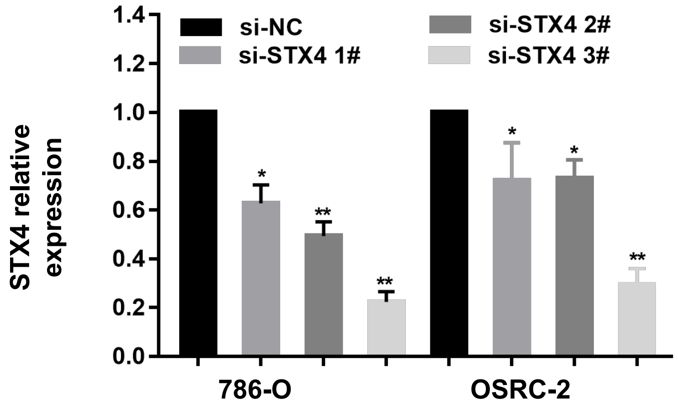

Figure S2: Validation of the knockdown effect of STX4 by qPCR

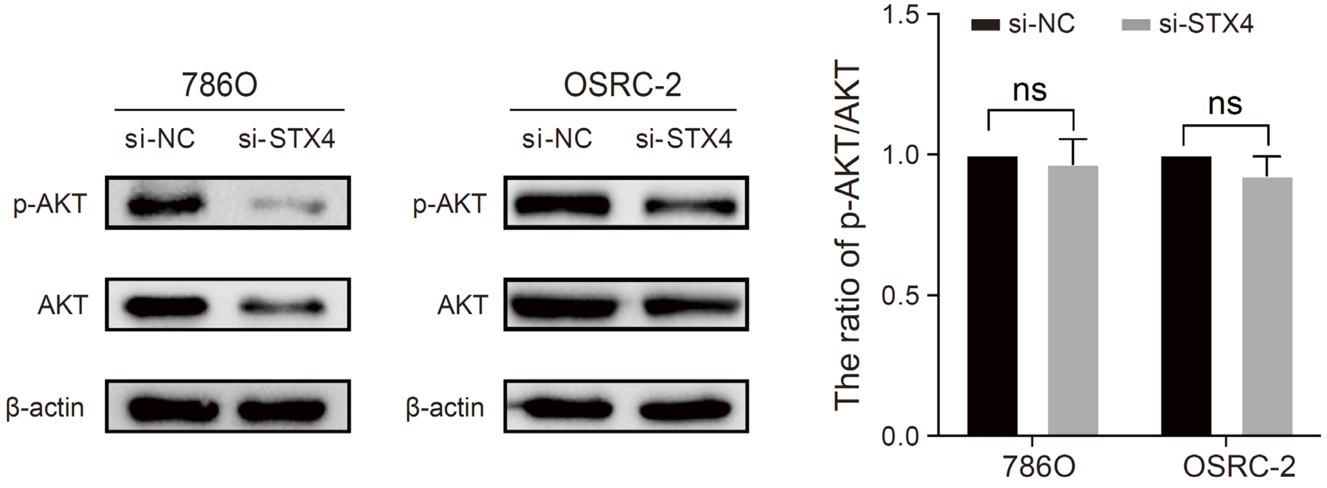

Figure S3: Western blot showed the expression of AKT and p-AKT in the si-NC and si-STX4 groups in 786O and OSRC-2 cell lines. There is no statistically significant disparity in the ratio of p-AKT and AKT between the two groups.

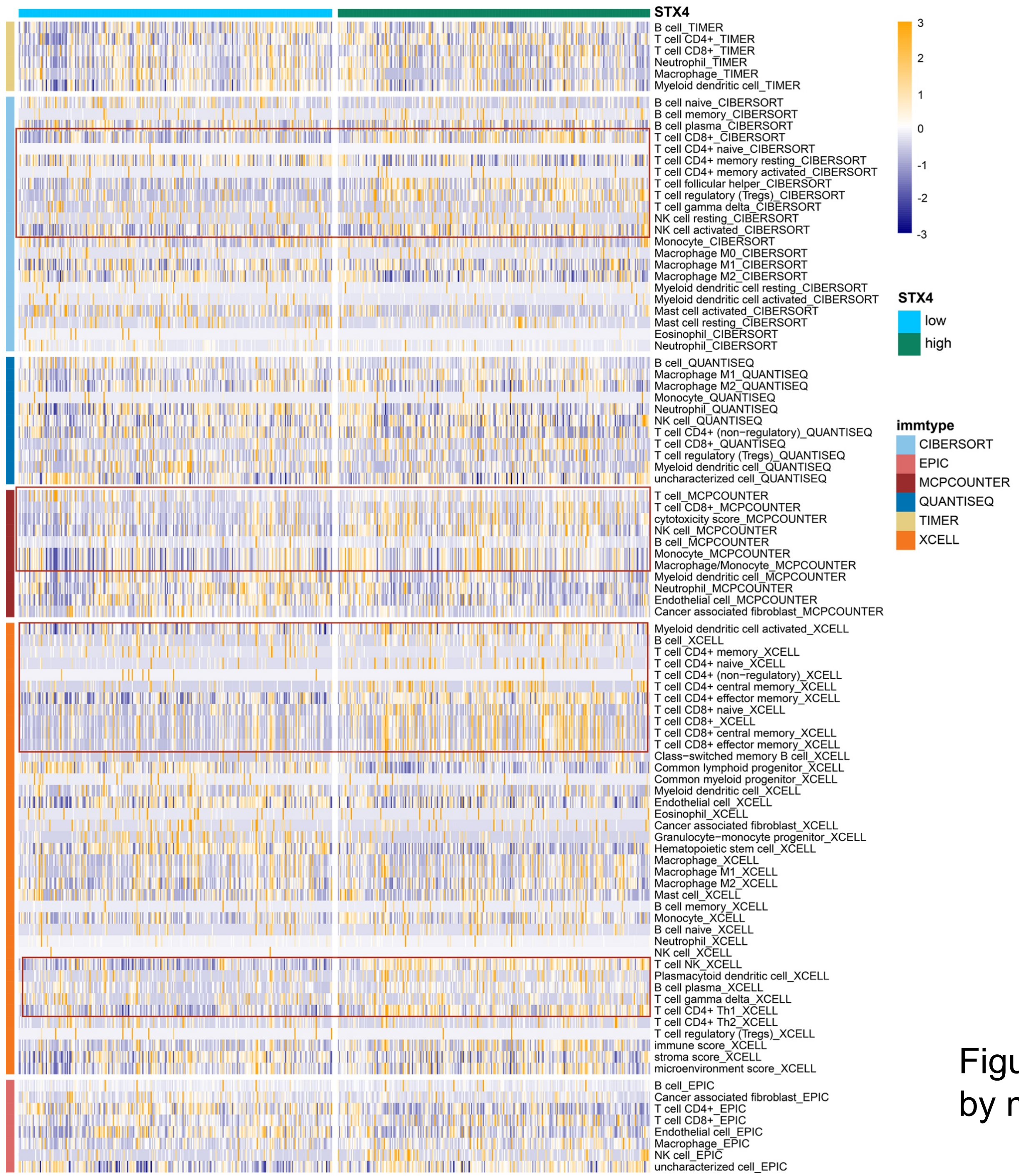

Figure S4: Heatmap of immune cells infiltration level predicted by multiple algorithms in the STX4^{high} and STX4^{low} group.

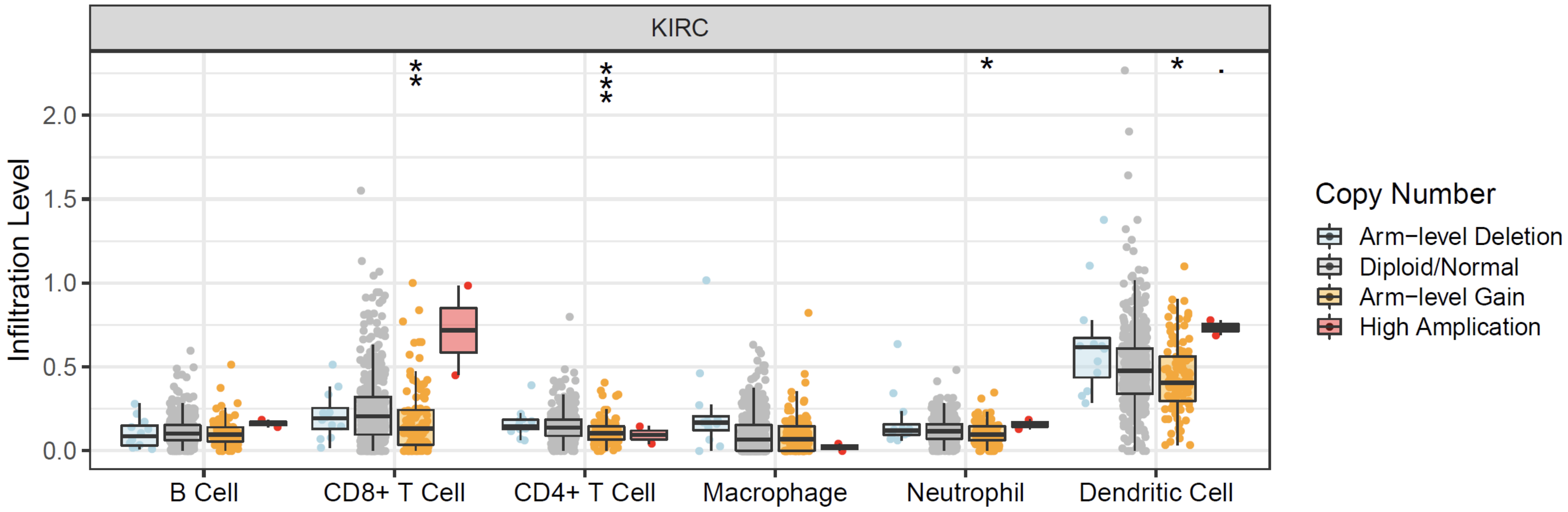

Figure S5: Association between STX4 gene copy number and immune cells infiltration level.

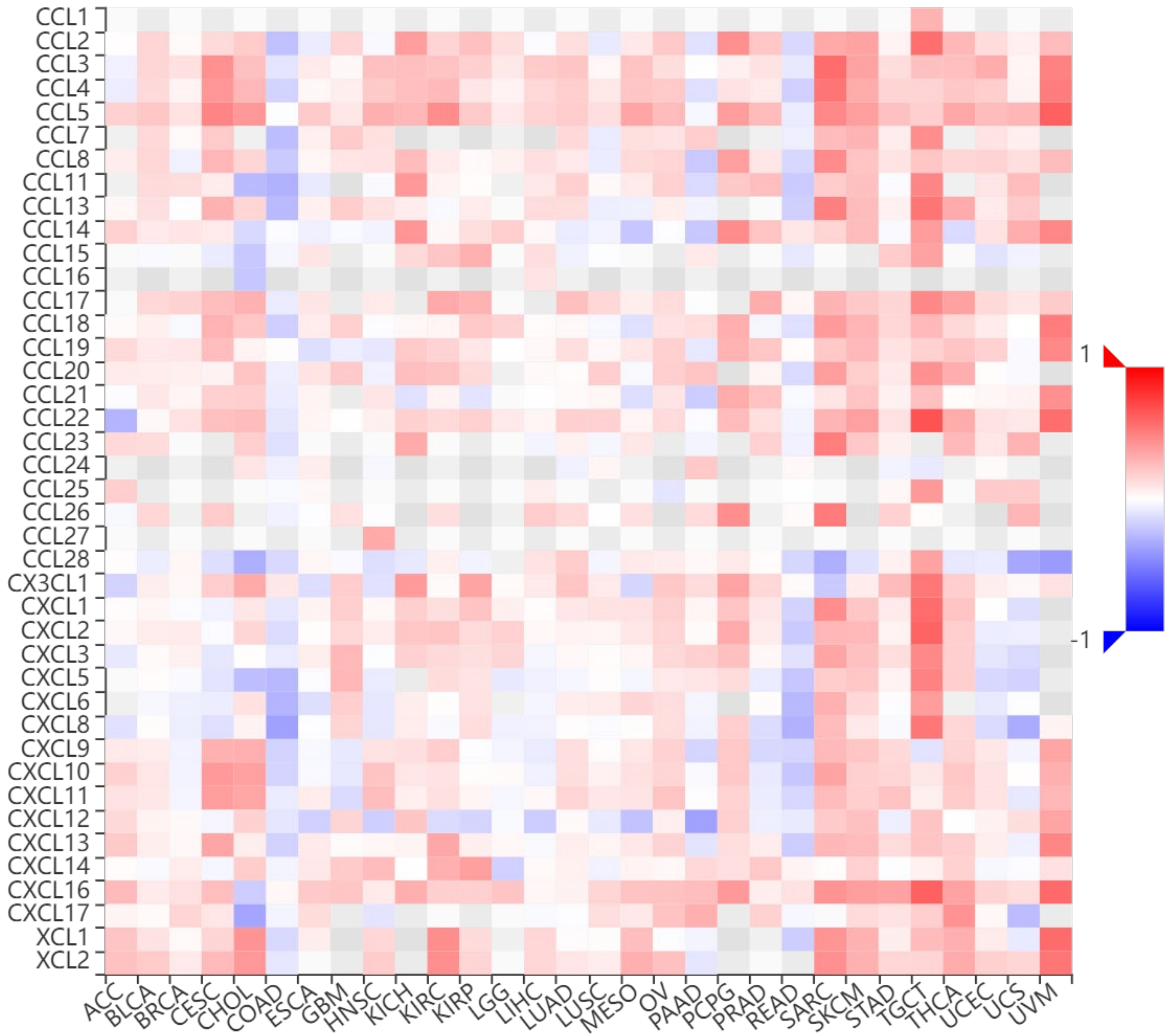

Figure S6: Correlations between chemokines and STX4 expression in various cancer types.

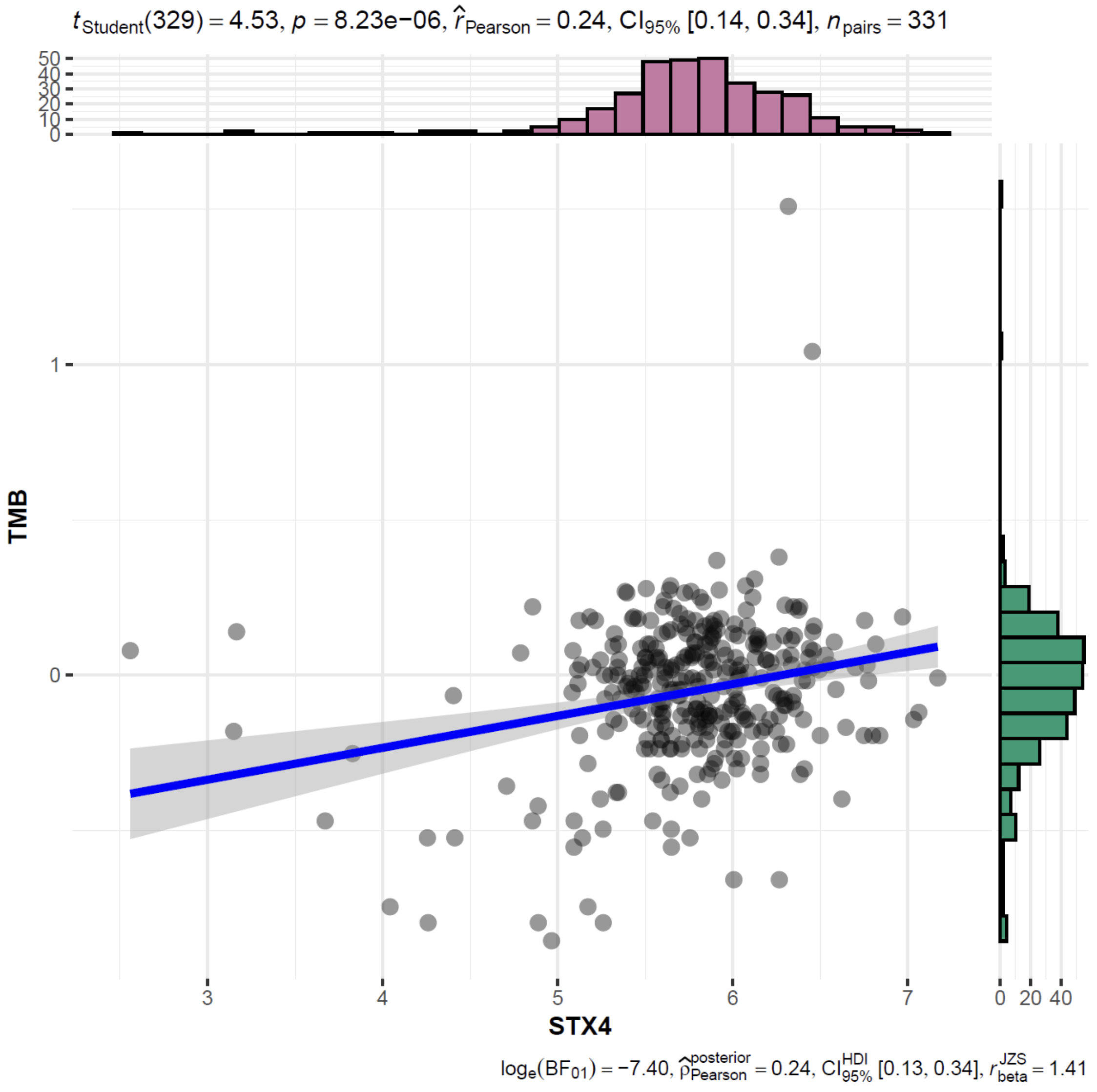

Figure S7: Correlation analysis of STX4 expression and TMB.

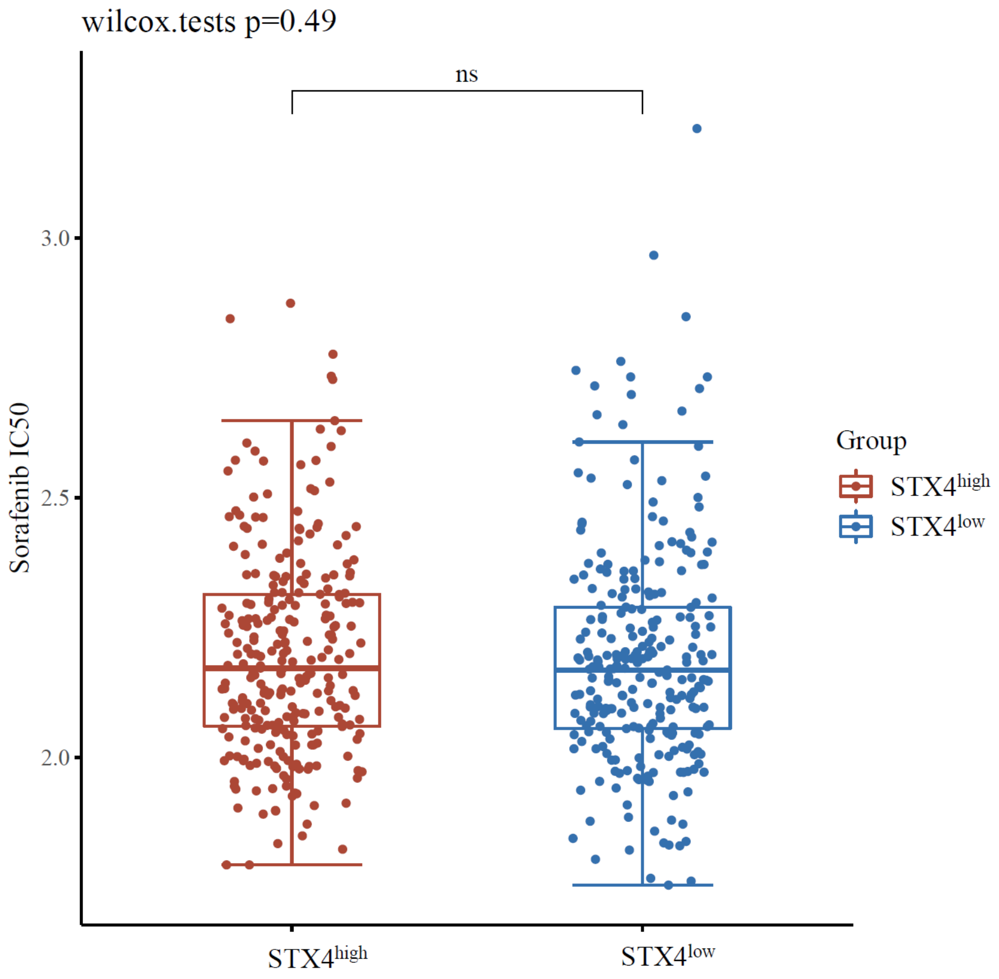

Figure S8: Predicted IC50 of sorafenib between STX4^{high} and STX4^{low} group.

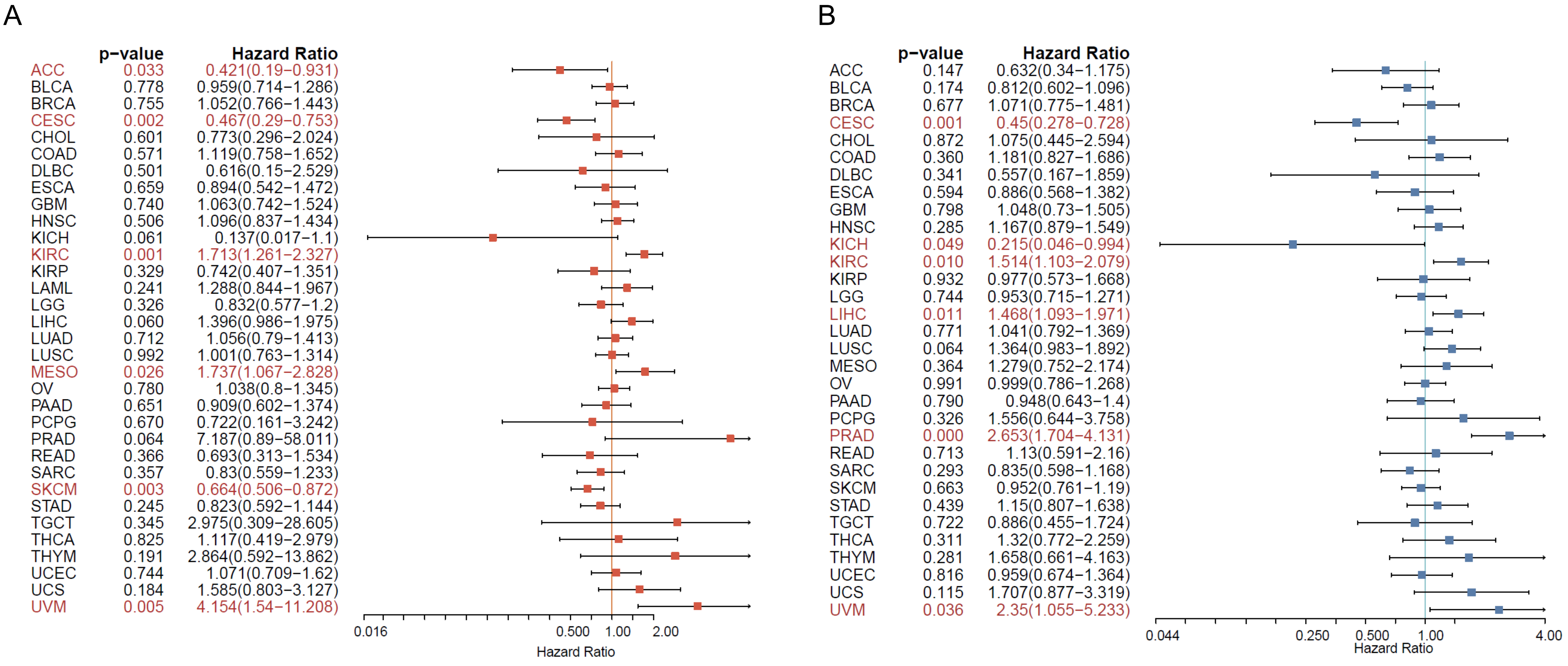

Figure S9: Association between OS (A), PFS (B) and STX4 expression in pan cancers.
