# Supplementary material for: STX4 as a potential biomarker for predicting prognosis and guiding clinical treatment decisions in clear cell renal cell carcinoma
Source: Heliyon. 2023 Dec 21;10(1):e23918. doi: 10.1016/j.heliyon.2023.e23918 (PMC10788513; doi:10.1016/j.heliyon.2023.e23918)
Supplement: Multimedia component 3 [file mmc3.docx]

**Abbreviations**

CAFs: Cancer-associated fibroblasts

ccRCC: Clear cell renal cell carcinoma

DSS: Disease-specific survival

ECM: Extracellular matrix

GDSC: Genomics of Drug Sensitivity in Cancer

GEO: Gene Expression Omnibus

GO: Gene Ontology

HLA: Human leukocyte antigen

HPA: The Human Protein Atlas

ICB: Immune checkpoint blockade

IC50: Half maximal inhibitory concentration

IPS: Immunophenoscore

KEGG: Kyoto Encyclopedia of Genes and Genomes

KICH: Kidney Chromophobe

KIRP: Kidney renal papillary cell carcinoma

MDSCs: Myeloid-derived suppressor cells

MSI: Microsatellite Instability

OS: Overall survival

PFS: Progression-free survival

RCC: Renal cell carcinomas

SNARE: soluble N-ethylmaleimide-sensitive factor attachment protein receptors

ssGSEA: Single sample gene set enrichment analysis

TAMs: Tumor-associated macrophages

TCGA: The Cancer Genome Atlas

TICA: The Cancer Immunome Database

TIDE: Tumor Immune Dysfunction and Exclusion

TMB: Tumor mutation burden

TME: Tumor microenvironment

**Website**:

TCGA: <https://portal.gdc.cancer.gov/>

GEO: <https://www.ncbi.nlm.nih.gov/geo/>

TIMER 2.0: <http://timer.cistrome.org/>

GDSC database: <https://www.cancerrxgene.org/>

TIDE: <http://tide.dfci.harvard.edu/>

TCIA: <https://www.tcia.at/home>

CellMiner database: <https://discover.nci.nih.gov/cellminer/>

LinkedOmics database: <http://www.linkedomics.org/admin.php>

TISIDB database: <http://cis.hku.hk/TISIDB/index.php>

HPA database: <https://www.proteinatlas.org/>
