# Supplementary material for: STX4 as a potential biomarker for predicting prognosis and guiding clinical treatment decisions in clear cell renal cell carcinoma
Source: Heliyon. 2023 Dec 21;10(1):e23918. doi: 10.1016/j.heliyon.2023.e23918 (PMC10788513; doi:10.1016/j.heliyon.2023.e23918)
Supplement: Multimedia component 4 [file mmc4.pdf]

Figure 3A

786O

All Merge

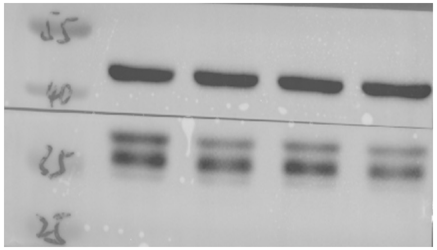

STX4

β-actin

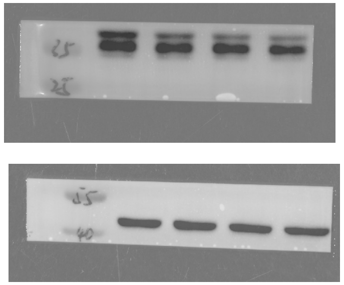

OSRC2

All Merge

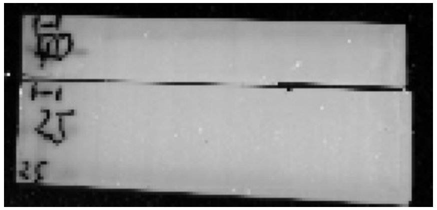

STX4

β-actin

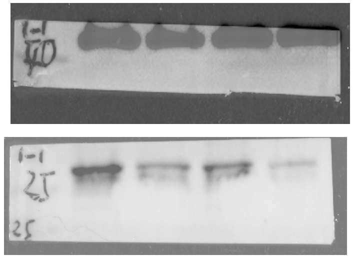

Figure 4C

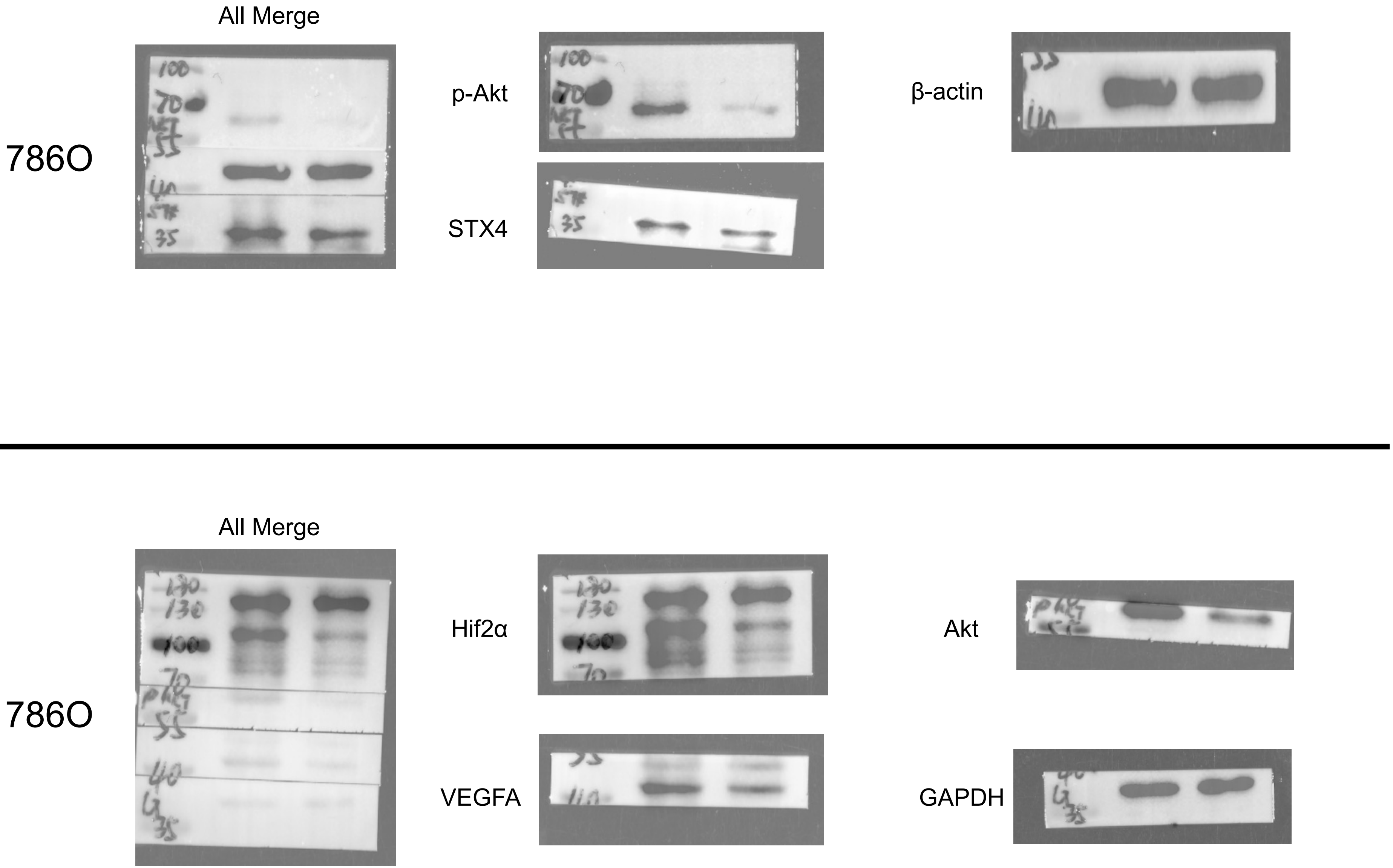
All Merge
786O
p-Akt
STX4
β-actin
All Merge
786O
Hif2α
VEGFA
Akt
GAPDH

Figure 4C

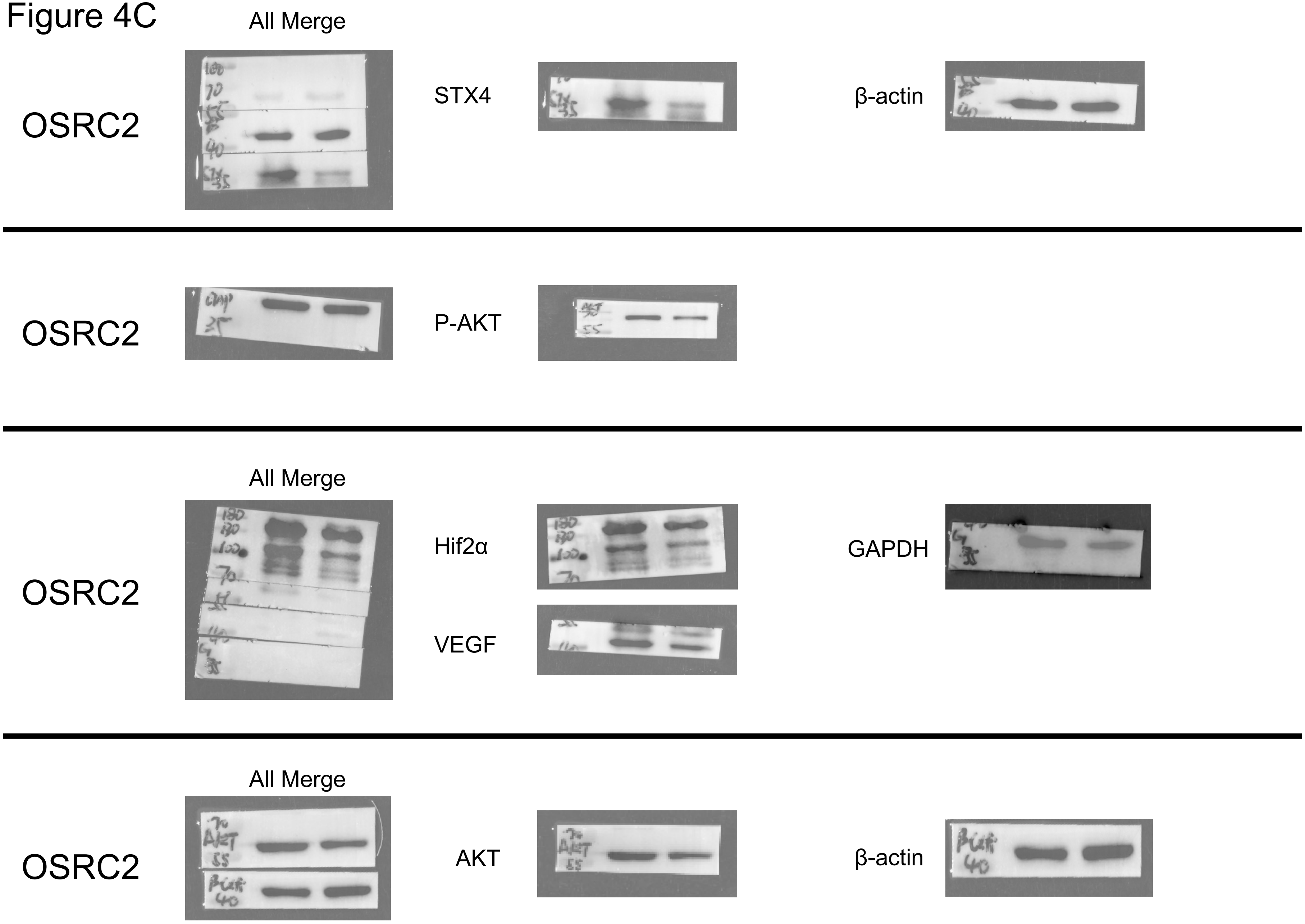
All Merge
OSRC2
STX4
β-actin
OSRC2
P-AKT
All Merge
OSRC2
Hif2α
VEGF
GAPDH
All Merge
OSRC2
AKT
β-actin
